# Cerebral metabolism is not affected by moderate hyperventilation in patients with traumatic brain injury

**DOI:** 10.1186/s13054-018-2304-6

**Published:** 2019-02-13

**Authors:** Giovanna Brandi, Nino Stocchetti, Alberto Pagnamenta, Federica Stretti, Peter Steiger, Stephanie Klinzing

**Affiliations:** 1 Institute for Intensive Care Medicine, University Hospital of Zurich, Rämistrasse 100, 8091 Zurich, Switzerland; 2 Department of Physiopathology and Transplantation, Milan University and Neuro ICU Fondazione IRCCSCà Granda Ospedale Maggiore Policlinico Milan, Milan, Italy; 3 Unit of Clinical Epidemiology, Ente Ospedaliero Cantonale, Bellinzona, Switzerland; 4 Department of Intensive Care, Westmead Hospital, Westmead, NSW 2145 Australia

**Keywords:** Traumatic brain injury, Hyperventilation, Intracranial pressure, Brain metabolites, Brain oxygenation, Transcranial color-coded duplex sonography

## Abstract

**Background:**

Hyperventilation-induced hypocapnia (HV) reduces elevated intracranial pressure (ICP), a dangerous and potentially fatal complication of traumatic brain injury (TBI). HV decreases the arteriolar diameter of intracranial vessels, raising the risk of cerebral ischemia. The aim of this study was to characterize the effects of moderate short-term HV in patients with severe TBI by using concomitant monitoring of cerebral metabolism, brain tissue oxygen tension (PbrO_2_), and cerebral hemodynamics with transcranial color-coded duplex sonography (TCCD).

**Methods:**

This prospective trial was conducted between May 2014 and May 2017 in the surgical intensive care unit (ICU) at the University Hospital of Zurich. Patients with nonpenetrating TBI older than 18 years of age with a Glasgow Coma Scale (GCS) score < 9 at presentation and with ICP monitoring, PbrO_2_, and/or microdialysis (MD) probes during ICU admission within 36 h after injury were included in our study. Data collection and TCCD measurements were performed at baseline (A), at the beginning of moderate HV (C), after 50 min of moderate HV (D), and after return to baseline (E). Moderate HV was defined as arterial partial pressure of carbon dioxide 4–4.7 kPa. Repeated measures analysis of variance was used to compare variables at the different time points, followed by post hoc analysis with Bonferroni adjustment as appropriate.

**Results:**

Eleven patients (64% males, mean age 36 ± 14 years) with an initial median GCS score of 7 (IQR 3–8) were enrolled. During HV, ICP and mean flow velocity (CBFV) in the middle cerebral artery decreased significantly. Glucose, lactate, and pyruvate in the brain extracellular fluid did not change significantly, whereas PbrO_2_ showed a statistically significant reduction but remained within the normal range.

**Conclusion:**

Moderate short-term hyperventilation has a potent effect on the cerebral blood flow, as shown by TCCD, with a concomitant ICP reduction. Under the specific conditions of this study, this degree of hyperventilation did not induce pathological alterations of brain metabolites and oxygenation.

**Trial registration:**

NCT03822026. Registered on 30 January 2019.

## Introduction

Hyperventilation-induced arterial hypocapnia (HV) has been used for decades in neuroanesthesia and neurointensive care, especially in the management of patients with severe traumatic brain injury (TBI) and concomitant elevated intracranial pressure (ICP). Increased alveolar ventilation induces a dose-response decrease in arterial partial pressure of carbon dioxide (PaCO_2_), causing a parallel reduction of carbon dioxide partial pressure in the extracellular brain compartment with tissue alkalosis. This results in vasoconstriction of the cerebral arterioles [1, 2]. Consequently, cerebral blood flow (CBF), cerebral blood volume (CBV), and ultimately ICP decrease.

The most relevant criticism of the use of HV is the risk of cerebral ischemia and hypoxia, especially in the acute phase of TBI [3]. This risk has been documented in several studies [4, 5], but the net effects of HV on regional CBF and metabolism are still debated [6].

Despite those concerns, HV is still widely used, even in the absence of elevated ICP and without brain oxygenation monitoring [7]. However, there is an international consensus that HV could have deleterious effects, especially by reducing CBF and brain oxygenation. To minimize this risk, HV should be used only to counteract the raised ICP, rather than prophylactically, and only with appropriate monitoring of CBF and/or oxygenation [8–11]. While continuous global measurements of CBF are not feasible at the bedside, cerebral blood flow velocity in the middle cerebral artery (CBFV) may be measured by transcranial Doppler and has been used to monitor the effects of hypocapnia [12, 13]. The present study was conducted to quantify potential adverse effects of moderate short-term HV during the acute phase of severe TBI on cerebral hemodynamics, oxygenation, and metabolism.

## Methods

The Institutional Ethics Committee of Zurich approved the research protocol of this prospective clinical trial (KEK-ZH 2012-0542). Informed consent was obtained from the patients’ next of kin prior to study enrollment and/or from the patients after ICU discharge.

### Patient population and initial stabilization

Inclusion criteria involved patients (≥ 18 years of age) with nonpenetrating head injury, with initial GCS score < 9 prior to sedation and intubation, extended neuromonitoring with ICP, PbrO_2_, and/or microdialysis probes, undergoing invasive mechanical ventilation with fraction of inspired oxygen < 60% and positive end-expiratory pressure < 15 cmH_2_O. Exclusion criteria were decompressive craniectomy, pregnancy, preexisting neurologic disease, previous TBI, acute cardiovascular disease, severe respiratory failure, acute or chronic liver disease, sepsis, and failure to obtain satisfactory bilateral TCCD signals. Patients with persisting hypovolemia or hemodynamic instability despite previous fluid resuscitation (defined as global end-diastolic volume index < 680 ml/m^2^, central venous oxygen saturation [ScvO_2_] < 60%, or increase in mean arterial blood pressure [MAP] > 15% after passive leg raise test) were excluded. All patients were treated according to a cerebral perfusion orientated protocol aiming to achieve cerebral perfusion pressure (CPP) > 70 mmHg, ICP ≤ 20 mmHg, and PbrO_2_ > 15 mmHg. PaCO_2_ was targeted at 4.8–5.2 kPa.

### Monitoring

MAP, ICP, CPP, arterial oxygen saturation (SaO_2_), end-tidal CO_2_ (etCO_2_), and PbrO_2_ were continuously monitored. ICP was measured with a fiberoptic device (Camino, San Diego, CA, USA) and PbrO_2_ with a Clark-type microcatheter (Licox GmbH, Kiel, Germany).

Cerebral microdialysis (MD) was performed using a catheter (CMA70; Microdialysis, Solna, Sweden) with a membrane length of 10 mm and a molecular mass cutoff of 20 kDa, which was perfused by a microinjection pump (CMA 106; Microdialysis) with artificial cerebrospinal fluid at a flow rate of 0.3 μl/min. Samples were collected every hour and immediately analyzed for glucose, lactate, and pyruvate with a bedside MD analyzer (CMA 600; CMA/Microdialysis). Sampling was performed at 1-h intervals in order to allow the detection of sufficient concentration of analytes in the microdialysate. ICP, PbrO_2_, and MD catheters were placed in the white frontal matter, usually on the side of the brain with more significant injury. The correct position of the catheters was confirmed by computed tomography.

TCCD examinations of the middle cerebral artery (MCA) were performed bilaterally through the transtemporal acoustic window by two experienced physicians (SK, GB) according to standard techniques [14, 15] using a 2-MHz Probe (Philips CX 50; Philips Healthcare, Andover, MA, USA). Three repeated measurements of the peak systolic velocity (PSV) and end-diastolic velocity (EDV) were performed for each side. The device automatically calculated CBFV and pulsatility index (PI). PI was chosen for its high predictive value in case of increased ICP and low CPP [16].

### Study protocol

**Fig. 1:**
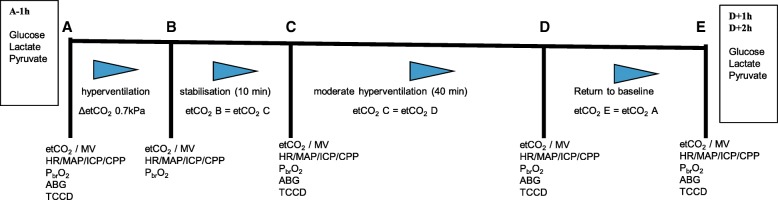
Study protocol. At different time points, several variables were collected and transcranial color-coded duplex sonography (TCCD) measurements were performed. **A** = Baseline. **B** = Increasing minute ventilation. **C** = Begin moderate hyperventilation with target PaCO_2_ 4–4.7 kPa. **D** = After moderate hyperventilation for 50 min. **E** = Return to baseline. Glucose, lactate, and pyruvate concentrations in the extracellular fluid were sampled by microdialysis 1 h before A (A − 1 h) and 1 h (D + 1 h) and 2 h after D (D + 2). *etCO*_*2*_ End-tidal CO_2_ (kPa), *MV* Minute ventilation (L/min), *HR* Heart rate (beats/min), *MAP* Mean arterial pressure (mmHg), *ICP* Intracranial pressure (mmHg), *CPP* Cerebral perfusion pressure (mmHg), *PbrO*_*2*_ Brain tissue oxygen tension (mmHg), *ABGA* Arterial blood gas analysis

Patients were enrolled in the study within 36 h of sustaining trauma. Under baseline conditions, a TCCD examination was performed, and all parameters were recorded (Fig. 1, point A). The alveolar ventilation was then increased over a 10-min period to obtain moderate HV by a stepwise increase in tidal volume and respiratory rate until a reduction of etCO_2_ of 0.7 kPa (Fig. 1, point B) was achieved.

After 10 min of stable etCO_2_, a second TCCD measurement was made (beginning of HV; Fig. 1, point C). The etCO_2_ value was kept stable for 40 min, followed by a third TCCD examination (Fig. 1, point D). Finally, normoventilation was reestablished over 10 min, and all variables were allowed to return to baseline (Fig. 1, point E). A final TCCD examination was conducted at this time point. At each time point, MAP, ICP, CPP, PbrO_2_, SpO_2_, and etCO_2_ were recorded.

Microdialysate samples for glucose, lactate, and pyruvate concentrations were collected 1 h before A (A − 1 h) as a baseline measurement and 1 h (D + 1 h) and 2 h (D + 2 h) after D to detect possible changes induced by moderate HV. Arterial blood gas tests (ABG) were obtained at points A, C, D, and E to monitor the changes of pH and PaCO_2_.

### Statistical analysis

Data were analyzed to produce mean ± SD or median with IQR, unless otherwise indicated. Repeated-measures analysis of variance (ANOVA) was used to compare all variables. When the *F*-ratio of the ANOVA reached a critical level (corresponding to *p* < 0.05), post hoc analysis with Bonferroni adjustment was used. All tests were performed two-sided, and *p* < 0.05 was considered statistically significant. Statistical analysis was performed using Stata version 12.1 software (StataCorp LP, College Station, TX, USA).

## Results

### Demographic data

**Fig. 2:**
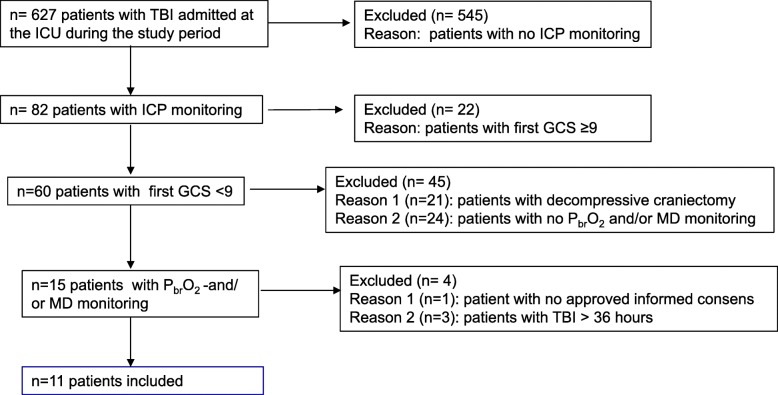
Flowchart of the study. During the study period, 627 patients were admitted with a diagnosis of traumatic brain injury (TBI). Reasons for exclusion from the study are shown on the right side of the chart. *ICU* Intensive care unit, *ICP* intracranial pressure, *GCS* Glasgow Coma Scale, *PbrO*_*2*_ Brain tissue oxygen tension, *MD* Cerebral microdialysis

During the study period, 628 patients with TBI were admitted to the surgical ICU. A breakdown of reasons for exclusion is presented in Fig. 2. Eleven patients with severe TBI were included; 64% were males, with a mean age of 36 ± 14 years. The median first GCS score was 7 (IQR 3–8). The admission computed tomographic (CT) scan identified several brain lesions, very often combined. An epidural hematoma was diagnosed in three patients (27%), a subdural hematoma in seven (64%), contusions in eight (73%), and edema/swelling in three (27%).

On average, the Injury Severity Score (ISS) was 31 ± 11, and the Simplified Acute Physiology Score (SAPS) II 24 h after TBI was, on average, 48 ± 9. The study was performed, on average, 23 h after TBI.

### Ventilation parameters

Changes in PaCO_2_ during the test and other respiratory variables are shown in Table 1.

**Table 1:** Respiratory variables

*n* = 11	A Baseline	B Minute ventilation	C Moderate hyperventilation	D 50 min of moderate hyperventilation	E Return to baseline
etCO_2_ (kPa)	5.0 (0.7)	4.3 (0.6)*	4.2 (0.6)*^§^	4.0 (0.7)*^§^	4.7 (0.7)*^§¶Ŧ^
MV (L/min)	6.9 (1.4)	8.6 (1.6)*	8.7 (1.7)*	8.4 (1.5)*	6.8 (1.4)^§¶Ŧ^
pH	7.37 (0.09)		7.45 (0.02)*	7.46 (0.03)*^¶^	7.41 (0.03)^¶Ŧ^
PaCO_2_ (kPa)	5.0 (0.2)		4.3 (0.2)*	4.1 (0.4)*^¶^	4.7 (0.4)*^¶Ŧ^
PaO_2_ (kPa)	17.2 (1.5)		17.9 (1.9)	18.8 (2.3)*^¶^	17.4 (1.2)^Ŧ^

*Abbreviations: A* Baseline, *B* Increasing minute ventilation, *C* Begin moderate hyperventilation with target PaCO_2_ 4–4.7 kPa, *D* After moderate hyperventilation for 50 min, *E* Return to baseline, *etCO*_*2*_ End-tidal CO_2_, *MV* Minute ventilation (L/min), *PaCO*_*2*_ Partial pressure arterial carbon dioxide (kPa), *PaO*_*2*_ Partial pressure arterial oxygen (kPa)

Data are expressed as mean (SD)

* *p* < 0.05 compared with A

^§^ *p* < 0.05 compared with B

^¶^ *p* < 0.05 compared with C

^Ŧ^ *p* < 0.05 compared with D

### Effects of hyperventilation on systemic and cerebral hemodynamics

MAP and HR (Table 2) did not change significantly during the study. Mean ICP was 16 ± 6 mmHg (A), decreased significantly to 8 ± 6 mmHg (*p* < 0.0001) during C, and remained stable during D (10 ± 7 mmHg; *p* = 0.30). On return to normoventilation (E), ICP increased significantly to 14 ± 6 mmHg (*p* = 0.003 compared with D) and was similar to A (*p* = 0.06).

**Table 2:** Systemic and cerebral hemodynamics

*n* = 11	A Baseline	B Minute ventilation	C Moderate hyperventilation	D 50 min of moderate hyperventilation	E Return to baseline
HR (beats/min)	72 (16)	72 (17)	71 (17)	71 (17)	66 (16)
MAP (mmHg)	92 (10)	92 (10)	93 (12)	90 (10)	94 (13)
ICP (mmHg)	16 (6)	8 (5)*	8 (6)*	10 (7)*	14 (6)^§¶Ŧ^
CPP (mmHg)	77 (9)	84 (9)*	85 (10)*	81 (14)	80 (11)^§¶^
PbrO_2_ (mmHg)	32 (10)	33 (9)	33 (10)	30 (8)^§¶^	30 (8)^§^

*Abbreviations: A* Baseline, *B* Increasing minute ventilation, *C* Begin moderate hyperventilation with target PaCO_2_ 4–4.7 kPa, *D* After moderate hyperventilation for 50 min, *E* Return to baseline, *HR* Heart rate (beats/min), *MAP* Mean arterial pressure (mmHg), *ICP* Intracranial pressure (mmHg), *CPP* Cerebral perfusion pressure (mmHg), *PbrO*_*2*_ Brain tissue oxygen tension (mmHg)

*N* = 11 patients; + = 10 patients. Data are expressed as mean (SD)

* *p* < 0.05 compared with A

§ *p* < 0.05 compared with B

¶ *p* < 0.05 compared with C

Ŧ *p* < 0.05 compared with D

### Effects of hyperventilation on mean flow velocity and PI in the middle cerebral artery

TCCD measurements in the right and left MCAs were analyzed separately. CBFV was 81 ± 23 cm/s and 80 ± 22 in the right and left MCAs, respectively. During C, CBFV decreased to 69 ± 21 cm/s in the right MCA (*p* = 0.0002) and to 66 ± 17 cm/s in the left MCA (*p* = 0.006). CBFV did not change significantly during D (71 ± 20 cm/s, *p* = 0.38, and 63 ± 15 cm/s, *p* = 0.48, in the right and left MCA, respectively) compared with C. During E, CBFV was 77 ± 28 cm/s (*p* = 0.21, compared with D) and 75 ± 24 cm/s (*p* = 0.005, compared with D) at the right and left sides, respectively.

PI was 1.03 ± 0.17 and 0.96 ± 0.15 in the right and left MCAs, respectively. During the study, PI did not change significantly in the right MCA (Table 3). In the left MCA, PI remained stable during C (1.06 ± 0.10, *p* = 0.056, compared with A), increased significantly during D (1.07 ± 0.14, *p* < 0.03, compared with A), and remained stable during E (1.02 ± 0.14, *p* = 0.12, compared with D).

**Table 3:** Transcranial color-coded duplex sonography variables measured in middle cerebral arteries bilaterally

*n* = 11	A Baseline	C Moderate hyperventilation	D 50 min of moderate hyperventilation	E Return to baseline
CBFV right (cm/s)	81 (23)	69 (21)*	71 (20)*	77 (28)
CBFV left (cm/s)	80 (22)	66 (17)*	63 (15)*	75 (24)^¶Ŧ^
PSV right (cm/s)	136 (37)	117 (34)*	120 (33)*	129 (42)
PSV left (cm/s)	131 (35)	110 (25)*	107 (24)*	126 (37)^¶Ŧ^
EDV right (cm/s)	56 (20)	45 (16)*	46 (16)*	51 (22)^¶^
EDV left (cm/s)	54 (16)	43 (13)*	41 (11)*	52 (17)^¶Ŧ^
PI right	1.03 (0.17)	1.08 (0.16)	1.05 (0.23)	1.05 (0.17)
PI left	0.96 (0.15)	1.06 (0.10)	1.07 (0.14)*	1.02 (0.14)

*Abbreviations: A* Baseline, *B* Increasing minute ventilation, *C* Begin moderate hyperventilation with target PaCO_2_ 4–4.7 kPa, *D* After moderate hyperventilation for 50 min, *E* Return to baseline. *CBFV* Mean flow velocity of the middle cerebral artery (cm/s), *PSV* Peak systolic velocity (cm/s), *EDV* End-diastolic velocity (cm/s), *PI* Pulsatility index

* *p* < 0.05 compared with A

¶ *p* < 0.05 compared with C

Ŧ *p* < 0.05 compared with D

*N* = 11 patients, + = 10 patients. Data are expressed as mean (SD)

Changes of PSV and EDV on both sides are shown in Table 3.

### Brain tissue oxygen tension and cerebral metabolism

PbrO_2_ and MD were measured in 10 and 9 patients, respectively. Eight patients got PbrO_2_ and MD simultaneously, and both probes were placed close to each other. The parenchyma surrounding the probes appeared normal on the CT scan in nine patients, seven of them with both PbrO_2_ and MD monitoring, one with only MD monitoring, and one with only PbrO_2_. In two cases, the parenchyma surrounding the probes appeared pathological; one of them had PbrO_2_ and MD probes inserted, and one only a PbrO_2_ probe.

Mean PbrO_2_ was 32 ± 10 mmHg; it remained stable during C (33 ± 10 *p* = 0.50), decreased during D (30 ± 8 *p* = 0.003, compared with C), and remained stable during E (30 ± 8 *p* = 0.90 compared with D) (Table 2).

The mean extracellular fluid concentrations of glucose, lactate, pyruvate, and the lactate/pyruvate ratio in the brain 1 h before and 1 and 2 h after moderate HV are reported in Table 4. No significant changes were observed.

**Table 4:** Cerebral microdialysis

*n* = 9	Baseline (A − 1 h)	1 h after moderate hyperventilation for 50 min (D + 1 h)	2 h after moderate hyperventilation for 50 min (D + 2 h)
Brain glucose (mmol/L)	1.5 (1.0)^Φ^	1.4 (0.7)	1.4 (0.7)
Brain lactate (mmol/L)	3.3 (1.0)^Φ^	3.5 (1.3)	3.4 (1.4)
Brain pyruvate (μmol/L)	101.4 (38.3)^Φ^	99.6 (39.5)	97.3 (44.7)^Φ^
LP ratio	34.2 (8.2)^Φ^	39.0 (17.7)	37.5 (11.4)^Φ^

*LP* Lactate/pyruvate ratio

Data are expressed as mean (SD). *N* = 9 patients

^Φ^ Eight patients

## Discussion

### Main findings

The aim of this study was to detect possible adverse effects of moderate HV in patients with severe TBI during the acute phase postinjury. To our knowledge, this is the first study using a multimodal monitoring set (MD, PbrO_2_, and TCCD, together with ICP and CPP) to investigate this.

Our data suggest that moderate short-term HV was effective in lowering ICP with a remarkable effect on CBF velocities, as shown by the reduction of the CBFV in the MCAs bilaterally and by a slight increase in PI. The CBFV in the MCAs in the study population at baseline was normal, suggesting a physiological global CBF. In the course of moderate HV, CBFV and hence CBF decreased significantly, as in previous studies [17, 18]. These findings are consistent with vasoconstriction in the brain vasculature leading to a reduction in CBF and likely in CBV.

MAP and HR remained stable during moderate HV, as demonstrated by Minhas et al. [13] in volunteers. However, in patients receiving positive pressure ventilation, sedation, and hypovolemia, the interactions between hypocapnia and hemodynamics are more complex [2]. Due to the possible systemic effects of hypocapnia, patients with persisting hypovolemia or hemodynamic instability despite previous fluid resuscitation were excluded.

Our work included two additional variables, PbrO_2_ and MD, with the specific aim of demonstrating possible tissue hypoxia and/or disturbed metabolic patterns.

On the basis of our results, there was a PbrO_2_ reduction, but within a physiological range. Additionally, MD with a stable Lactate/pyruvate (L/P) ratio was not indicative of an energy crisis.

#### What is already known

In the last 30 years, HV has been fiercely debated. After initial widespread use [19], the effects of HV on outcome were questioned in a randomized study [20]. That study included a total of 113 patients in 3 groups and used different levels of HV in all arms. ICP was kept stable in all groups. At 3 and 6 months after injury, patients with a motor score of 4–5 in the hyperventilation group had less favorable outcomes than the other groups. However, the control group was hyperventilated and had a PaCO_2_ of 31–32 mmHg for the first 5 study days. Additionally, HV was induced prophylactically, regardless of ICP.

More accurate studies of pathophysiological changes induced by HV have been performed by two authoritative groups. Coles et al. measured a significant reduction of CBF with PET scans and an increase in the volume of severely hypoperfused tissue following HV [4] with a PaCO_2_ reduction from 4.8 to 3.9 kPa. Diringer et al., on the contrary, using an analogous PET technique, did not disclose any alteration of oxygen metabolism in two groups of patients with TBI managed with different degrees of HV [17]. They concluded that oxygen metabolism was maintained due to the low baseline metabolic rate and compensatory increase in oxygen extraction fraction.

#### How our data compare with the literature: limitations

Our data questioned if HV, even when effective in reducing CBF and ICP, induced measurable changes compatible with true ischemia (defined as a CBF insufficient for preserving adequate tissue oxygenation and metabolism). For a fair comparison with the existing evidence, however, the study limitations should be acknowledged. First, the small sample size limits the generalizability of the findings. More accurate analyses, for instance looking at sex differences in vasoreactivity or association between PI and outcome, were clearly not feasible [21, 22]. Second, we decided to perform only a moderate degree of short-term HV. This choice was dictated by two concerns: the risk of ischemia and the time frame of the test. We were aware that the brain is especially vulnerable to ischemia during the acute phase after trauma [23]. It may well be that we did not detect pathological changes because of the mild level of hypocapnia used for a short time interval. Third, the MD and PbrO_2_ probes explore only small areas of the brain; therefore, we cannot exclude concomitant changes in other brain regions [24, 25].

In our study population, however, the MD and the PbrO_2_ probes were placed, in the majority of the cases, in the most injured hemisphere of the brain. Interestingly, the cerebral glucose at baseline was normal, whereas the L/P ratio at baseline was markedly increased and pathological [26], reflecting tissue injury despite “normal” parenchyma on the CT scan. From this point of view, we were exploring a vulnerable portion of the brain, where potential side effects of HV would have been more likely to be detected. Furthermore, because none of the probes were placed directly in the site of contusion and only two were in pericontusional tissue, the effect of HV on PbrO_2_ should not have been compromised [24].

## Conclusions

Our findings add evidence to the belief that a moderate degree of short-term HV is effective in reducing ICP through CBFV modifications as an estimation of CBF. Furthermore, a moderate degree of HV reduces PbrO_2_, but within a physiological range, and does not significantly change the cerebral energy metabolism. Further investigations of prolonged and more severe HV, in the context of intracranial hypertension and in a larger study population, are needed to characterize the risks and benefits of HV in severe TBI.
